# Editorial: Advances in Photoacoustic Neuroimaging

**DOI:** 10.3389/fnins.2022.859515

**Published:** 2022-03-07

**Authors:** Biqin Dong, Junjie Yao, Xosé Luís Deán-Ben

**Affiliations:** ^1^Academy for Engineering and Technology, Fudan University, Shanghai, China; ^2^Department of Biomedical Engineering, Duke University, Durham, NC, United States; ^3^Institute for Biomedical Engineering, Faculty of Medicine, University of Zurich, Zurich, Switzerland; ^4^Institute of Pharmacology and Toxicology, Faculty of Medicine, University of Zurich, Zurich, Switzerland; ^5^Institute for Biomedical Engineering, Department of Information Technology and Electrical Engineering, ETH Zurich, Zurich, Switzerland

**Keywords:** photoacoustics, neuroimaging, functional brain imaging, optical absorption contrast, optical ultrasound sensors, image reconstruction and processing algorithms

In recent years, rapid progress in laser technology, ultrasound detectors, imaging agents, animal models, as well as reconstruction and processing methods have significantly advanced photoacoustic (optoacoustic) neuroimaging, promoting its ability in investigating brain morphology, function, and biochemical composition at multiple spatial and temporal scales (Yao and Wang, 2014). This Research Topic summarizes these recent advances in photoacoustic neuroimaging and their impact on our understanding of the nervous system and brain diseases.

The review article contributed by Bodea and Westmeyer highlights the current capabilities of photoacoustic techniques for preclinical research in neuroscience based on small rodents. The ability to visualize anatomical structures based on contrast derived from exogenous and endogenous photoabsorbers makes photoacoustic imaging a valuable tool for the non-invasive monitoring of neoplastic diseases, such as brain tumors. Due to its sensitivity to blood dynamics and hemoglobin oxygenation (Roberts et al., 2018), functional photoacoustic imaging can be used to monitor pathological brain conditions and is naturally suited for studying brain ischemia, epileptic seizures, and neurodegenerative diseases through investigating cerebral hemodynamic responses associated with injury, disease, and aging. Together with rapid volumetric photoacoustic imaging, the development of novel photoacoustic contrast agents and dynamic molecular sensors is expected to provide robust signal changes in response to activation events in deep brain. This will enable a potent platform to directly record and modulate neuronal activity and further broaden the capabilities and impact of this technology in neuroscience. The review article further discussed emerging opportunities for translation of photoacoustic neuroimaging into the clinical setting (Steinberg et al., 2019), which could accelerate rapid and safe diagnosis of the margin of resection during brain surgery, diabetic neuropathy, neonates' brain injury, etc, as well as provide technical possibilities to label-free intraoperative neuroimaging and miniaturized photoacoustic endoscopy for lumbar puncture or needle-based biopsies.

One main obstacle for photoacoustic neuroimaging in both small animals and humans is the skull, which introduces strong distortion and attenuation of both excitation light and the generated ultrasound waves. The quality of the photoacoustic brain images can be significantly deteriorated, thus craniotomy is still timely performed to optimize the achievable resolution and overall image quality (Manwar et al., 2020). Optical imaging of the brain is often assisted with optical clearing agents and transparent cranial windows to avoid repeated surgeries in longitudinal studies. In a similar manner, skull clearing agents have demonstrated their effectiveness in *in vivo* photoacoustic neuroimaging experiments (Yang et al., 2016). An alternative approach is to use a transparent cranial window capable of ultra-sensitive ultrasound sensing for photoacoustic brain imaging (Li et al., 2019). Such a cranial window can be surgically implanted on the skull to establish an optimal acoustic coupling condition while maintaining optical access. In this Research Topic, a convenient approach based on a long-term hidden cranial window in a mouse is proposed by Wang et al. By removing the skull and suturing the incision on the scalp, transcranial ultrasound blockage is effectively eliminated with minimum physiological perturbation. The effectiveness of the new model in both photoacoustic and ultrasound brain imaging was confirmed with the observed increase in signal strength and image quality.

The quality of the photoacoustic images of the brain further relies on the operational performance of the ultrasonic transducer employed to detect the photoacoustic signals. As a well-established technology widely used in photoacoustic imaging, traditional piezoelectric transducers have some shortcomings that restrict the further development of photoacoustics, particularly for brain imaging, and often cause technical difficulties in integrating photoacoustic imaging with conventional optical imaging modalities (Lin et al., 2021). Recently, optical ultrasound sensors have been suggested for photoacoustic imaging. These provide a broadband frequency response, wide angular coverage, a small footprint, and potential optical transparency (Dong et al., 2016; Wissmeyer et al., 2018). These features attract growing attention of employing optical ultrasound sensors in photoacoustic brain imaging. For this instance, a novel all-optical ultrasound sensor based on a Fabry-Pérot polymer cavity for imaging *in vivo* mouse brain structure and function with high resolution is contributed by Chen et al. The sensor's cavity length is dynamically tuned by a beam of heating light, thus enabling the resonant wavelength to be quickly adjusted to lock the sensor at its maximal sensitivity. Such a tuning mechanism enables sensor interrogation at a single wavelength, leading to the reduced overall cost and potential multiplexing capability.

Image reconstruction and processing algorithms are also essential to maximize the amount of information that can be extracted from photoacoustic brain images. Particularly, multi-spectral (multi-wavelength) imaging is essential to resolve dynamic changes in oxygenated and deoxygenated hemoglobin induced via neurovascular coupling, as well as calcium signals directly visualizing neuronal activity (Gottschalk et al., 2019). Advanced processing of the acquired sequence of images is then essential in photoacoustic neuroimaging. However, image reconstruction represents a more fundamental step for accurate quantification. Photoacoustic images are generally reconstructed with back-projection algorithms similar to delay-and-sum methods used in ultrasound imaging. Model-based (iterative) algorithms have further been developed to provide more quantitative images (Rosenthal et al., 2013). These algorithms enabled e.g., compressed-sensing-based image reconstruction from partial data, which significantly enhanced the achievable temporal resolution (Özbek et al., 2018). More recently, machine- and deep-learning-based methods have been shown to provide unprecedented performance in photoacoustic image reconstruction and processing. In this Research Topic, a new deep-learning approach is introduced by Shahid et al. to effectively eliminate undersampling artifacts. A comparison of multiple neural networks revealed that high-quality photoacoustic images can be reconstructed with only 5% of the original fully-sampled data acquired with a standard system.

The articles included in this Research Topic are a sample of the strong momentum of photoacoustic neuroimaging. It is foreseeable that future advances in photoacoustic neuroimaging depend as much on the close collaboration among optical engineers, chemists, synthetic biologists, materials scientists, and data scientists to facilitate further innovations. This is expected to make photoacoustic neuroimaging a more powerful tool for preclinical and clinical applications in neurophysiology and neuropathology.

## Author Contributions

All authors listed have made a substantial, direct, and intellectual contribution to the work and approved it for publication.

## Conflict of Interest

The authors declare that the research was conducted in the absence of any commercial or financial relationships that could be construed as a potential conflict of interest.

## Publisher's Note

All claims expressed in this article are solely those of the authors and do not necessarily represent those of their affiliated organizations, or those of the publisher, the editors and the reviewers. Any product that may be evaluated in this article, or claim that may be made by its manufacturer, is not guaranteed or endorsed by the publisher.

## References

[B1] Dong B. Sun C. Zhang H. F. (2016). Optical detection of ultrasound in photoacoustic imaging. IEEE Trans. Biomed. Eng. 64, 4–15. 10.1109/TBME.2016.2605451 27608445 PMC5222629

[B2] Gottschalk S. Degtyaruk O. Mc Larney B. Rebling J. Hutter M. A. Deán-Ben X. L.. (2019). Rapid volumetric optoacoustic imaging of neural dynamics across the mouse brain. Nat. Biomed. Eng. 3, 392–401. 10.1038/s41551-019-0372-9 30992553 PMC6825512

[B3] Li H. Dong B. Zhang X. Shu X. Chen X. Hai R.. (2019). Disposable ultrasound-sensing chronic cranial window by soft nanoimprinting lithography. Nat. Commun. 10, 1–9. 10.17605/OSF.IO/DT8VE 31537800 PMC6753120

[B4] Lin L. Hu P. Tong X. Na S. Cao R. Yuan X.. (2021). High-speed three-dimensional photoacoustic computed tomography for preclinical research and clinical translation. Nat. Commun. 12, 1–10. 10.1038/s41467-021-21232-1 33563996 PMC7873071

[B5] Manwar R. Kratkiewicz K. Avanaki K. (2020). Investigation of the effect of the skull in transcranial photoacoustic imaging: a preliminary ex vivo study. Sensors. 20, 4189. 10.3390/s20154189 32731449 PMC7435985

[B6] Özbek A. Deán-Ben X. L. Razansky D. (2018). Optoacoustic imaging at kilohertz volumetric frame rates. Optica 5, 857–863. 10.1364/OPTICA.5.000857 31608306 PMC6788779

[B7] Roberts S. Seeger M. Jiang Y. Mishra A. Sigmund F. Stelzl A.. (2018). Calcium sensor for photoacoustic imaging. J. Am. Chem. Soc. 140, 2718–2721. 10.1021/jacs.7b03064 28945084

[B8] Rosenthal A. Ntziachristos V. Razansky D. (2013). Acoustic inversion in optoacoustic tomography: A review. Curr. Med. Imag. 9, 318–336. 10.2174/15734056113096660006 24772060 PMC3996917

[B9] Steinberg I. Huland D. M. Vermesh O. Frostig H. E. Tummers W. S. Gambhir S. S. (2019). Photoacoustic clinical imaging. Photoacoustics 14, 77–98. 10.1016/j.pacs.2019.05.001 31293884 PMC6595011

[B10] Wissmeyer G. Pleitez M. A. Rosenthal A. Ntziachristos V. (2018). Looking at sound: optoacoustics with all-optical ultrasound detection. Light Sci. Appl. 7, 1–16. 10.1038/s41377-018-0036-7 30839640 PMC6107019

[B11] Yang X. Zhang Y. Zhao K. Zhao Y. Liu Y. Gong H.. (2016). Skull optical clearing solution for enhancing ultrasonic and photoacoustic imaging. IEEE Trans. Med. Imaging 35, 1903–1906. 10.1109/TMI.2016.2528284 26886977

[B12] Yao J. Wang L. V. (2014). Photoacoustic brain imaging: from microscopic to macroscopic scales. Neurophotonics 1, 011003. 10.1117/1.nph.1.1.011003 25401121 PMC4232215

